# Predictive models of long COVID

**DOI:** 10.1016/j.ebiom.2023.104777

**Published:** 2023-09-04

**Authors:** Blessy Antony, Hannah Blau, Elena Casiraghi, Johanna J. Loomba, Tiffany J. Callahan, Bryan J. Laraway, Kenneth J. Wilkins, Corneliu C. Antonescu, Giorgio Valentini, Andrew E. Williams, Peter N. Robinson, Justin T. Reese, T.M. Murali, N3C consortium, Christopher Chute

**Affiliations:** a Department of Computer Science, Virginia Polytechnic Institute and State University (Virginia Tech), Blacksburg, VA, 24061, USA; b The Jackson Laboratory for Genomic Medicine, Farmington, CT, 06032, USA; c AnacletoLab, Computer Science Department, Dipartimento di Informatica, Università degli Studi di Milano, Milan, 20133, Italy; d Division of Environmental Genomics and Systems Biology, Lawrence Berkeley National Laboratory, Berkeley, CA, 94720, USA; e ELLIS - European Laboratory for Learning and Intelligent Systems, Milan Unit, Milan, 20133, Italy; f Integrated Translational Health Research Institute of Virginia, University of Virginia, Charlottesville, VA, 22904, USA; g Department of Biomedical Informatics, Columbia University Irving Medical Center, New York, NY, 10032, USA; h Department of Biomedical Informatics, University of Colorado Anschutz Medical Campus, Aurora, CO, 80045, USA; i Biostatistics Program, Office of the Director, National Institute of Diabetes and Digestive and Kidney Diseases, National Institutes of Health, Bethesda, MD, 20814, USA; j Banner Health, University of Arizona, Phoenix, AZ, 85006, USA; k Institute for Clinical Research and Health Policy Studies, Tufts University School of Medicine, Boston, MA, 02111, USA; l Institute for Systems Genomics, University of Connecticut, Farmington, CT, 06269, USA

**Keywords:** Long COVID, COVID-19, Classification, Explainability, Cross-site analysis

## Abstract

**Background:**

The cause and symptoms of long COVID are poorly understood. It is challenging to predict whether a given COVID-19 patient will develop long COVID in the future.

**Methods:**

We used electronic health record (EHR) data from the National COVID Cohort Collaborative to predict the incidence of long COVID. We trained two machine learning (ML) models — logistic regression (LR) and random forest (RF). Features used to train predictors included symptoms and drugs ordered during acute infection, measures of COVID-19 treatment, pre-COVID comorbidities, and demographic information. We assigned the ‘long COVID’ label to patients diagnosed with the U09.9 ICD10-CM code. The cohorts included patients with (a) EHRs reported from data partners using U09.9 ICD10-CM code and (b) at least one EHR in each feature category. We analysed three cohorts: all patients (*n* = 2,190,579; diagnosed with long COVID = 17,036), inpatients (149,319; 3,295), and outpatients (2,041,260; 13,741).

**Findings:**

LR and RF models yielded median AUROC of 0.76 and 0.75, respectively. Ablation study revealed that drugs had the highest influence on the prediction task. The SHAP method identified age, gender, cough, fatigue, albuterol, obesity, diabetes, and chronic lung disease as explanatory features. Models trained on data from one N3C partner and tested on data from the other partners had average AUROC of 0.75.

**Interpretation:**

ML-based classification using EHR information from the acute infection period is effective in predicting long COVID. SHAP methods identified important features for prediction. Cross-site analysis demonstrated the generalizability of the proposed methodology.

**Funding:**

10.13039/100006108NCATS U24 TR002306, 10.13039/100006108NCATS UL1 TR003015, Axle Informatics Subcontract: NCATS-P00438-B, 10.13039/100000002NIH/10.13039/100000062NIDDK/OD, PSR2015-1720GVALE_01, G43C22001320007, and Director, Office of Science, Office of Basic Energy Sciences of the 10.13039/100006120U.S. Department of Energy Contract No. DE-AC02-05CH11231.

**Research in context**

**Evidence before this study**

Studies in the literature estimate that 10–70% of Coronavirus Disease 2019 (COVID-19) patients may go on to develop post-acute sequelae of Severe acute respiratory syndrome coronavirus 2 (SARS-CoV-2) infection (PASC or long Coronavirus Disease (long COVID)) subsequent to their initial infection. However, standard definitions of long COVID are just beginning to emerge and are yet to be widely adopted in studies or clinical guidelines. Hence the prognosis of long COVID in COVID-19 patients is a challenging task. We searched PubMed for studies published during or after 2020 that proposed data-driven computational methods for long COVID prediction. There were two studies (PMID 33692530 and 35589549) that leveraged machine learning to identify potential long COVID patients. However these studies defined long COVID labels based on self-reported diagnoses or visits to specialised long COVID clinics, both of which are unreliable markers of the disease. Additionally, one of the studies trained models with EHR data from up to a year after the acute infection, which possibly contained signals relevant to long COVID. We did not identify any existing publications that used information only from the acute SARS-CoV-2 infection phase or the reliable U09.9 ICD10-CM code to predict the occurrence of long COVID in COVID-19 patients. Besides, there were no studies that evaluated the impact of disparate data sources on the performance of the prediction models.

**Added value of this study**

Unlike previous publications, we labelled patients diagnosed with U09.9 ICD10-CM code as long COVID patients and used only information from the acute SARS-CoV-2 infection to define features. Logistic regression and random forest models predicted the occurrence of long COVID in COVID-19 patients with high area under receiver-operating characteristic. The performance of models trained on data from one N3C partner and tested on data from the other partners was on par with the classifiers trained on data from all sources. This cross-site analysis provides suggestive evidence for the generalizability of the prognosis methodology proposed in this study.

**Implications of all the available evidence**

Machine learning models trained using information from the electronic health records of COVID-19 patients during the acute infection phase can effectively predict the future occurrence of long COVID and highlight informative predictors.

## Introduction

Acute Coronavirus Disease 2019 (COVID-19) is characterised by upper respiratory and systemic symptoms, and may be complicated by pneumonia, hyperinflammation, hypoxemic respiratory failure, a prothrombotic state, cardiac dysfunction, and kidney injury.^1^^,^^2^ Patients with COVID-19 have reported the persistence of cardiovascular, respiratory, psychiatric, and other heterogeneous symptoms including dyspnea, cough, chest pain, muscle pain, joint pain, headache, arthralgia, myalgia, fatigue, post-exertional malaise or poor endurance, fever, “brain fog” or cognitive impairment, paresthesia, insomnia, anosmia, dysgeusia, mood alterations, palpitations or tachycardia (which may be postural/orthostatic), lightheadedness, abdominal pain, diarrhoea, menstrual irregularities, altered sense of smell and/or taste, hair-loss, hoarse voice, and rash.^3^, ^4^, ^5^ Approximately 10–20% of COVID-19 patients may experience these protracted symptoms.^6^ Some of these symptoms persist for months or emerge after a delayed onset of several weeks.^7^, ^8^, ^9^ The diagnostic labels that refer to this long-term symptom pattern include “long Coronavirus Disease (COVID)” (used in this paper), “long-haul COVID”, “post-acute sequelae of Severe acute respiratory syndrome coronavirus 2 (SARS-CoV-2) infection (PASC)”, or “post COVID-19 condition” as named by the World Health Organization (WHO).^8^, ^9^, ^10^, ^11^, ^12^, ^13^, ^14^

Currently there is no widely accepted standard definition of the long COVID condition in terms of the symptoms that have developed or persisted, or the time period during which they are manifested.^5^^,^^7^^,^^8^^,^^12^^,^^13^^,^^15^, ^16^, ^17^, ^18^ Some patients with long COVID experienced only mild symptoms or were asymptomatic during the acute phase of infection. The diagnosis of long COVID is convoluted due to the lack of clarity about it.^14^ Thus, the definition of ground-truth labels and predicting whether a given COVID-19 patient will go on to develop long COVID in the future is a challenging task.^7^^,^^17^^,^^19^

There are several studies that analyse symptoms and risk factors associated with long COVID.^5^^,^^7^^,^^8^^,^^11^^,^^20^ There are two published prediction models that identify long COVID patients in a cohort of COVID-19 patients.^7^^,^^13^ The model by Sudre et al.^13^ relied on data entered by the users of a mobile application. Hence, its participants were not a representative sample of the COVID-19 patient population. In addition, the data is self-reported by the application users and may thus be prone to inaccuracies and inconsistencies. Pfaff et al.^7^ identified long COVID patients as those who sought care in specialised long COVID clinics at health institutions. However, there is no guarantee that these clinic visits may result in a long COVID diagnosis. In contrast, we identified long COVID patients using a more reliable marker — the “U09.9 (Post COVID condition, unspecified)” code introduced in 2022 into the widely accepted International Classification of Diseases, Tenth Revision, Clinical Modification (ICD-10-CM).^12^^,^^21^ As stipulated by the ICD-10-CM ontology, this code represents conditions related to COVID-19 such as chronic respiratory failure, loss of smell, loss of taste, multisystem inflammatory syndrome, pulmonary embolism, and pulmonary fibrosis during the post-COVID-19 infection period.^22^

Pfaff and colleagues^7^ drew features from an extensive time window — from a year before the index COVID-19 infection to a year after it. Thus, the patient data they used in their predictive model may have contained signals of long COVID. In order to maximise the clinical utility of prediction, it is imperative that a predictive model designed to identify COVID-19 patients with a high risk for long COVID, relies primarily on data gathered during the acute SARS-CoV-2 infection.^5^^,^^15^

**Fig. 1:**
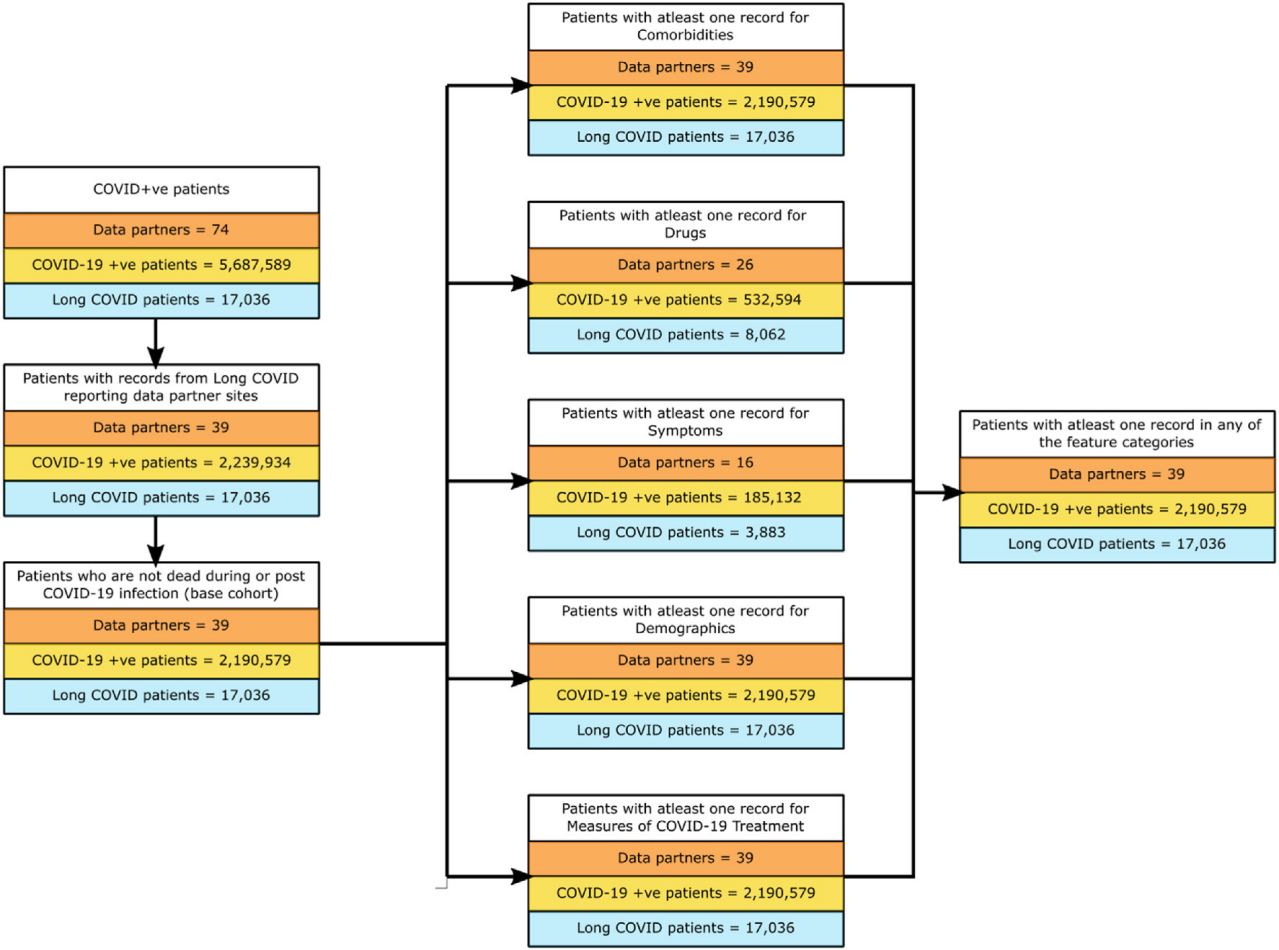
**Definition of all patient, inpatient, and outpatient cohorts**. The number of patients at each stage of the definition of the cohort of all patients. The dataset used for training and testing the prediction models consisted of 2,190,579 patients (data from 39 data partner sites) having at least one record in any of the five feature categories — comorbidities, drugs, symptoms, demographics, and measures of COVID-19 treatment. Of these COVID-19 positive patients, the number of long COVID patients, i.e., diagnosed with ICD-10-CM code U09.9, was 17,036.

In this cohort study, we leveraged pooled harmonised electronic health records (EHRs) from the National COVID Cohort Collaborative (N3C).^23^ The demographic information including age and gender of the patient base cohorts (Fig. 1, Methods: Patient Cohort Definition) is in Supplementary Table S1. We collected features during the acute phase of the disease (defined in our work as the 21-day period following the initial COVID-19 infection). Features included symptoms experienced by the patients, drugs ordered for them, measures of the treatment they received during their COVID-19 hospitalisation period (if applicable), patient demographics, and their comorbidities prior to COVID-19 infection. We implemented and compared the performance of logistic regression (LR) and random forest (RF) classification models in long COVID prognosis. These methods have been used extensively in medical applications.^24^^,^^25^ While LR is a simple, linear method, tree-based RF can capture nonlinear relationships between input features and target variables.

The principal contributions of this work are as follows — (ⅰ) We used the largest COVID-19 dataset to date to define a patient cohort that was reliably characterised and sufficiently large to support robust conclusions, and (ⅱ) rigorously defined long COVID diagnosis using only the U09.9 code. (ⅲ) While we trained machine learning (ML) models using balanced datasets, we used imbalanced test data to accurately reflect the prevalence of long COVID in patients and avoid incurring the bias induced by artificially balanced test sets.^26^ (ⅳ) We performed a cross-site analysis to validate the generalisability of our modelling framework across institutions. (ⅴ) We pooled interpretations of ML explanation techniques to explain the model predictions.

## Methods

### Data source

The N3C is an effort to collect, share, and analyse clinical data on COVID-19 in a secure and standardised manner.^23^ This EHR data comprises COVID-19 symptoms, health conditions, laboratory test results, medications, procedures administered to patients, information on patient mortality, and other observations and measurements such as blood pressure or oxygen saturation in arterial blood. It also includes demographic information such as age, sex, height and weight, race, and ethnicity. This database contains the largest cohort of COVID-19 cases in the United States^1^ including medical history dating back to January 2018.^27^ This data is hosted by the N3C Data Enclave and made available for public access through the secure data science platform Palantir Foundry.^23^ As of June 24, 2022, the N3C Data Enclave had records on 14.3 million people (including 5.6 million COVID positive cases) from 74 data partner sites.^28^ For this study, we used de-identified data in which each patient's dates of service are algorithmically shifted up to six months earlier or later than the true date, and ZIP codes are truncated to the first three digits.^29^ All dates in the EHRs pertaining to an individual are shifted consistently. Thus, the shifting does not compromise the acute infection phase defined in this study (Methods: Patient Cohort Definition, Symptoms during Acute COVID-19 Infection).

### Patient Cohort Definition

We created patient cohorts and labelled long COVID patients in a systematic manner from the N3C dataset as of June 24, 2022. Our COVID-19 positive population comprised patients who satisfied one or more of the following criteria: (ⅰ) a positive SARS-CoV-2 reverse transcription-polymerase chain reaction (RT-PCR) test, (ⅱ) a positive antigen test, or (ⅲ) a positive COVID-19 diagnosis, i.e., patients with at least one record with the ICD-10-CM code “U07.1 (COVID-19)”.^30^ This cohort had 5,687,589 patients across 73 data partner sites. However, only 39 data partner sites reported patients diagnosed with the ICD-10-CM U09.9 code, which corresponds to long COVID. After restricting our study to data reported by these 39 data partner sites, and excluding patients whose death had been recorded in the database (COVID-19 infection may not be the cause of death), the base population consisted of 2,190,579 patients. We subdivided the base population into three groups: (ⅰ) *inpatients*: patients who were hospitalised during the period starting from a day prior to the COVID-19 index date (the earliest date when that patient tested positive for COVID-19) to 16 days following the diagnosis date (*n* = 149,319), (ⅱ) *outpatients*: all other patients (*n* = 2,041,260), and (ⅲ) *all patients* (*n* = 2,190,579): the union of inpatient and outpatient groups. The final cohorts consisted of patients with at least one record in at least one of the five feature categories (Fig. 1, Supplementary Table S2 (Patient Counts); Methods: Feature Categories, Feature Combinations).

Five N3C data partner sites supplied information about patients visiting that site's local long COVID speciality clinic one or more times. However, a visit to such a clinic could be related to symptoms ultimately attributed to another medical condition that predated or followed COVID-19 infection. Since the symptoms of long COVID are highly non-specific, some long COVID clinics accept “self-referrals” in the absence of a prior medical assessment for long COVID, and long COVID is a diagnosis of exclusion. Thus, we elected to restrict the category label on which our algorithms were trained to those patients with a confirmed U09.9 diagnosis code (Yes = 1/No = 0) at institutions that had embraced the use of this code. Since this code initially came into use in October 2021, we acknowledge that our analysis has likely missed patients with long COVID that had been diagnosed prior to that time, but this bias was mitigated by having confined our analysis to centres that adopted use of this code. Therefore, we defined long COVID patients (positive samples, *n* = 17,036) as those having EHRs associated with the 2022 ICD-10-CM diagnosis code U09.9.^22^ All other patients from the base population were non-long COVID patients (negative samples, *n* = 2,173,543).

### Feature Categories

used a diverse set of features to train models to predict the occurrence of long COVID. They belonged to the following five categories: comorbidities, symptoms during acute COVID-19 infection, drugs, demographics, and measures of COVID-19 treatment (Supplementary Table S2 (Feature Counts)). Unless mentioned, every feature is categorical. Exceptions include numerical features such as age at the time of COVID-19 diagnosis and length of stay in hospital for COVID-19 treatment.

#### Comorbidities

This set of features included the conditions listed in the Charlson Comorbidity Index^31^ and the preexisting medical conditions identified by the Centers for Disease Control and Prevention (CDC) as making patients prone to a severe SARS-CoV-2 infection.^32^ Each feature had a value of 1 if the patient had a condition or observation related to the comorbidity on or prior to the COVID-19 index date; otherwise, the value was 0 (Supplementary Table S3 (Comorbidities)).

#### Symptoms during acute COVID-19 infection

The second set of features we considered were symptoms experienced by a patient after the COVID-19 diagnosis and during the acute infection phase. We defined this phase differently for inpatients and for outpatients.^8^ For an outpatient, we defined it as starting at the COVID-19 index date and ending at 21 days after this date. We used the same duration for an inpatient as well, unless the patient was treated in the hospital for more than 21 days; in this case, we ended the phase on the discharge date. This definition excluded symptoms experienced by a patient long after the acute infection phase, which may be a manifestation of long COVID.^8^

The symptoms feature category is different from the comorbidities category. Symptoms capture the conditions experienced by a patient during the acute infection phase, whereas comorbidities were conditions that a patient had at any time on or before the COVID-19 index date. A feature identified in the symptoms category may or may not be a comorbidity. The intent was to separate the existing health conditions in COVID-19 patients from the symptoms experienced by them during the acute infection.

In the N3C, Observational Medical Outcomes Partnership (OMOP) standard condition concepts are encoded using the Systemized Nomenclature of Medicine (SNOMED) vocabulary. We used mappings between OMOP and Open Biomedical Ontologies (OMOP2OBO) to map the SNOMED concepts to the Human Phenotype Ontology (HPO).^33^ Translating the disease conditions to HPO terms may help to better analyse long COVID.^8^ For each HPO term and patient, we assigned a value of 1 if the patient experienced the symptom corresponding to that HPO term during the acute infection phase; else the value was 0 (Supplementary Table S3 (Symptoms)).

#### Drugs

These features indicate the drugs ordered for the patients during the acute infection phase. We did not take into account why a drug is recorded in the EHRs, thus allowing any drug ordered or consumed for the treatment of a pre-existing condition or for SARS-CoV-2 infection to be considered as a feature. The harmonisation of vocabularies in N3C causes the same drug consumed in different dosages, administered through different methods, or manufactured and sold under different brand names to be recorded as separate concepts or entities. Thus, we grouped the drugs based on their active ingredients information in the N3C. A patient had a value of 1 for a drug group if there existed at least one drug record for that patient, during the acute COVID-19 infection, corresponding to any of the drugs mapped to that group, and otherwise a value of 0 (Supplementary Table S3 (Drugs)).

#### Demographics

Demographic information about a patient included age at the time of COVID-19 diagnosis and gender. While the age of the patient is a single numeric feature, gender was represented using multiple binary valued features using one-hot encoding. We used a feature called “Gender–Unknown” to record patients whose gender was not available (Supplementary Table S3 (Demographics)).

#### Measures of COVID-19 treatment

This set of features corresponded to aspects of the COVID-19 treatment for inpatients during the hospitalisation for COVID-19. These included the length of stay in the hospital and indicators to state whether Intermittent Mandatory Ventilation (IMV), Extracorporeal membrane oxygenation (ECMO), Remdesivir (the drug) was administered to the patients (Supplementary Table S3 (Measures of COVID-19 Treatment)).

### Feature Combinations

We constructed multiple datasets involving 15 different combinations of the feature categories described above for each of the three patient cohorts (Supplementary Method: Ablation Study Dataset Construction, Supplementary Table S2 (Ablation Study Feature Counts)). We trained and tested long COVID prediction models on each of these datasets and analysed the importance of different types of features in building a robust classifier.

### Model training, evaluation, and interpretation

**Fig. 2:**
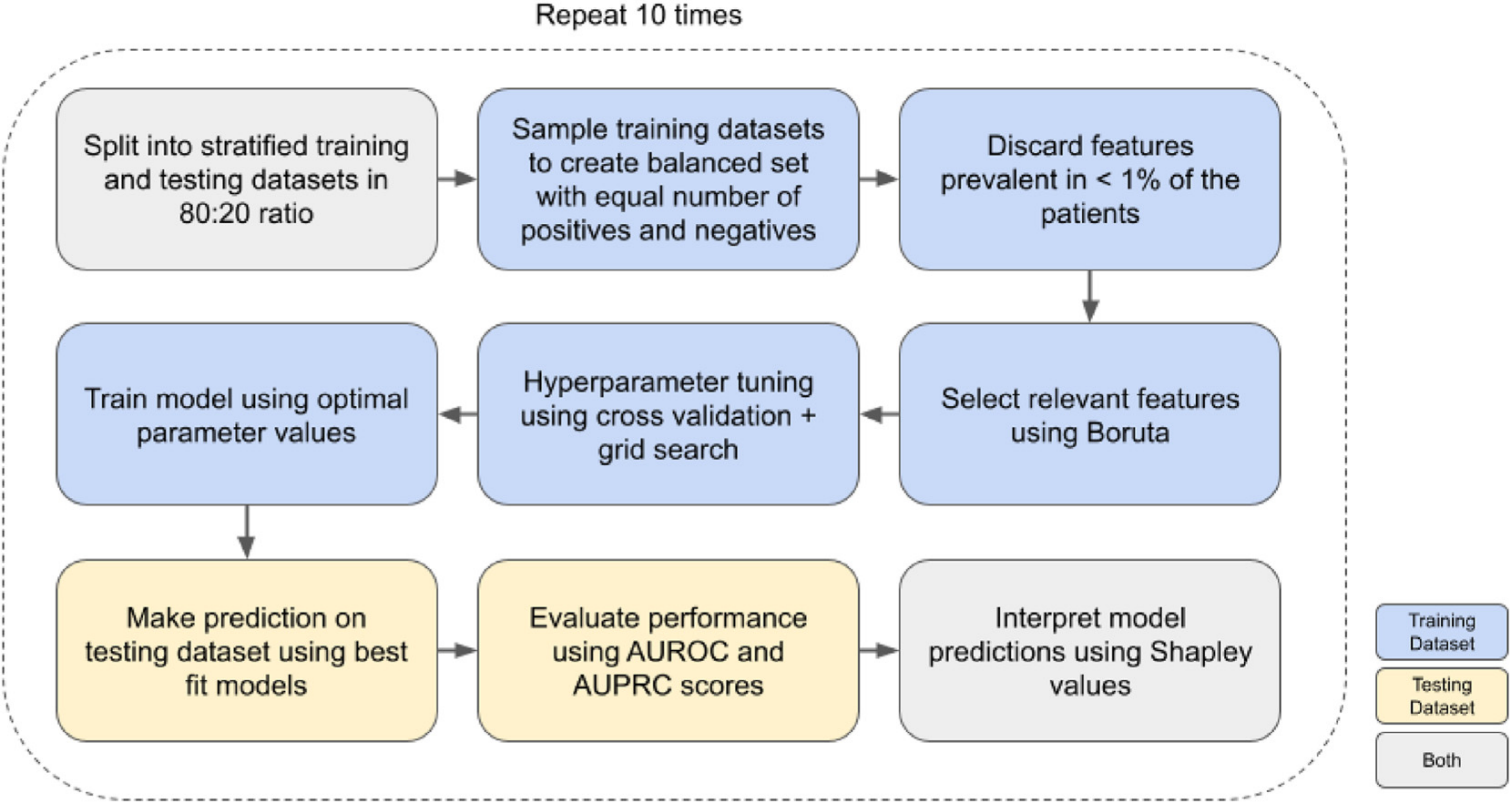
**Long COVID prediction pipeline**. Overview of the classification pipeline implemented for the prediction of long COVID.

Fig. 2 illustrates our complete pipeline for predicting long COVID in COVID-19 patients. We trained and evaluated LR and RF models independently using each of the three patient cohorts and the features defined above. Each experiment performed in this study involved ten (hold-out) iterations of the pipeline shown in Fig. 2.

#### Stratified hold-out

In each iteration, we used stratified hold-outs to obtain an unbiased evaluation. We used 80% of every dataset for training and the remaining 20% samples for testing (evaluation). The ratio of positive to negative samples in the training and test datasets was the same as in the original dataset. Since the all-patient cohort was highly skewed with only 0.78% positive samples (Fig. 1), we subsampled the training dataset to have almost equal number of positive and negative samples to reduce the risk of overfitting. However, we did not rebalance the testing dataset.

#### Feature selection

Due to the heterogeneity in the data, many features were present in a small set of patients. Examples of such rarely occurring features were Down's syndrome before COVID (prevalence = 0.02%), neck pain during the acute COVID-19 infection (0.10%), tuberculosis before COVID (0.20%), and oxytocin (0.26%).

To avoid any potential bias induced by such features, we used the training dataset to implement two levels of filtering. First, we used only those features present (i.e., there were patients associated with the feature) in at least 1% of the patient cohort. We settled on this threshold of 1% through trial and error to avoid filtering a large number of features in the very first stage. Second, we used the Boruta feature selection algorithm to select all the features that were relevant^34^ for the long COVID prediction task (Supplementary Table S4 (Prevalence and Boruta)). In every iteration, we applied the Boruta method on the training split to select features which we then used to train and test the prediction models in that particular iteration. Note that Boruta method may select different subsets of features in every iteration. Among the 86 features for all patients, Boruta algorithm selected a median of 38 features across the ten iterations. We narrowed down the features in the inpatient and outpatient cohorts using the same selection process.

#### Hyperparameter selection

We performed grid search^35^ using the training dataset to find the optimal hyperparameter values that maximised accuracy in a nested five-fold cross validation^36^ (Supplementary Table S5 (Hyperparameter Search)). We retrained the classifier, with the chosen hyperparameter values, on the entire training dataset and tested on the imbalanced, unseen testing dataset.

#### Evaluation

We compared the performance of the models trained on balanced datasets based on their ability to classify imbalanced testing datasets (Supplementary Table S2 (Dataset Splits)). We computed the area under receiver operating characteristic curve (AUROC) and the area under precision–recall curve (AUPRC) and reported the median and inter-quartile range (IQR) over the ten iterations.

#### Explanation and interpretation

We used the SHAP (SHapely Additive exPlanations)^37^^,^^38^ method to interpret each model prediction. This technique uses game theoretic principles to compute, for every patient, the contribution of each feature toward the prediction for the patient. Further, for each iteration, we computed the mean absolute value of the SHAP values of every feature over all test set samples in that iteration. We used these aggregated local interpretations to explain the overall model.

To draw further interpretable insights from the predictions, we analysed the SHAP values of individual test examples sampled randomly from the set of true positives, false positives, true negatives, and false negatives (Supplementary Methods: SHAP interpretation analysis) for the LR model.

### Statistics

In the ablation study, we trained models with different combinations of feature categories. We compared the AUROC scores over ten iterations of the LR and RF models on datasets with and without drug features using the Wilcoxon signed-rank test. For both models, non-parametric statistical tests yielded p-value <0.0001.

In the cross-site analysis, we used the Wilcoxon Rank Sum test to compare the AUROC scores of the models trained on EHRs from only data partner 1, only data partner 2, and from all data partners. The testing dataset included all remaining data samples in the final patient cohort. This non-parametric statistical test compared the statistical significance of the difference between two or more sets of populations. We observed varying p-values for different combinations of comparisons of the three populations. We performed this test for all patients, inpatients, and outpatients (Supplementary Table S8 (Statistical Test Results)).

### Role of funding sources

The funding sources did not have any role in study design, data collection, data analyses, interpretation, or writing of the report.

### Ethics

Participating institutions transfer electronic health records to the National Center for Advancing Translational Sciences (NCATS) under the Health Insurance Portability and Accountability Act (HIPAA). The data transfer is performed under a Johns Hopkins University Reliance Protocol #IRB00249128 or individual site agreements with National Institutes of Health (NIH). The N3C maintains this data in the N3C Data Enclave. Related information is available at https://ncats.nih.gov/n3c/about.

## Results

### Long COVID prediction

**Fig. 3:**
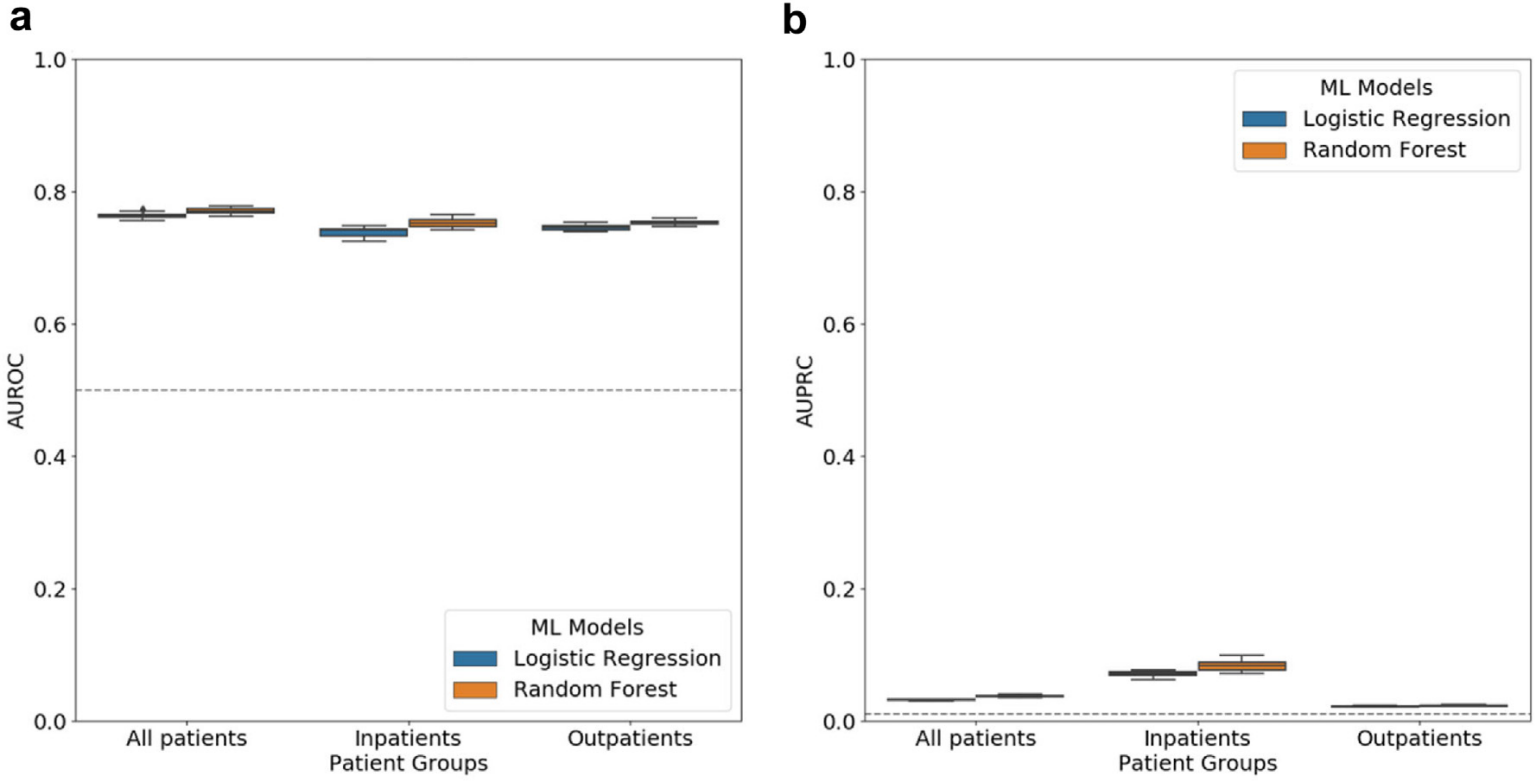
**Evaluation of long COVID prediction models in all three patient cohorts**. Distribution of **(a)** AUROC and **(b)** AUPRC scores from ten iterations of long COVID classification using logistic regression and random forest models for all patients, inpatients and outpatients. In each boxplot, the lower endpoint, the line in the middle, and the higher endpoint denote the first, second, and third quartiles of the distribution. The whiskers span 1.5 times the interquartile range. Diamonds denote values outside this range. The grey dotted line represents the expected score of a random predictor in the all-patient cohort.

For each classifier (LR and RF), we trained and tested ten separate models (ten iterations) using the three cohorts (all patients, inpatients, and outpatients) curated using five categories of features. LR and RF had virtually the same performance in all three cohorts (Fig. 3(a), Supplementary Figure S1, Supplementary Table S6 (AUROC)) with median AUROC and IQR between 0.74 (IQR = 0.01) and 0.77 (IQR = 0.01), and median AUPRC and IQR between 0.02 (IQR = 0.00) and 0.08 (IQR = 0.01) (Fig. 3(b), Supplementary Table S6 (AUPRC)). Both models yielded lowest AUROC and AUPRC scores in outpatients.

### Ablation Study

**Fig. 4:**
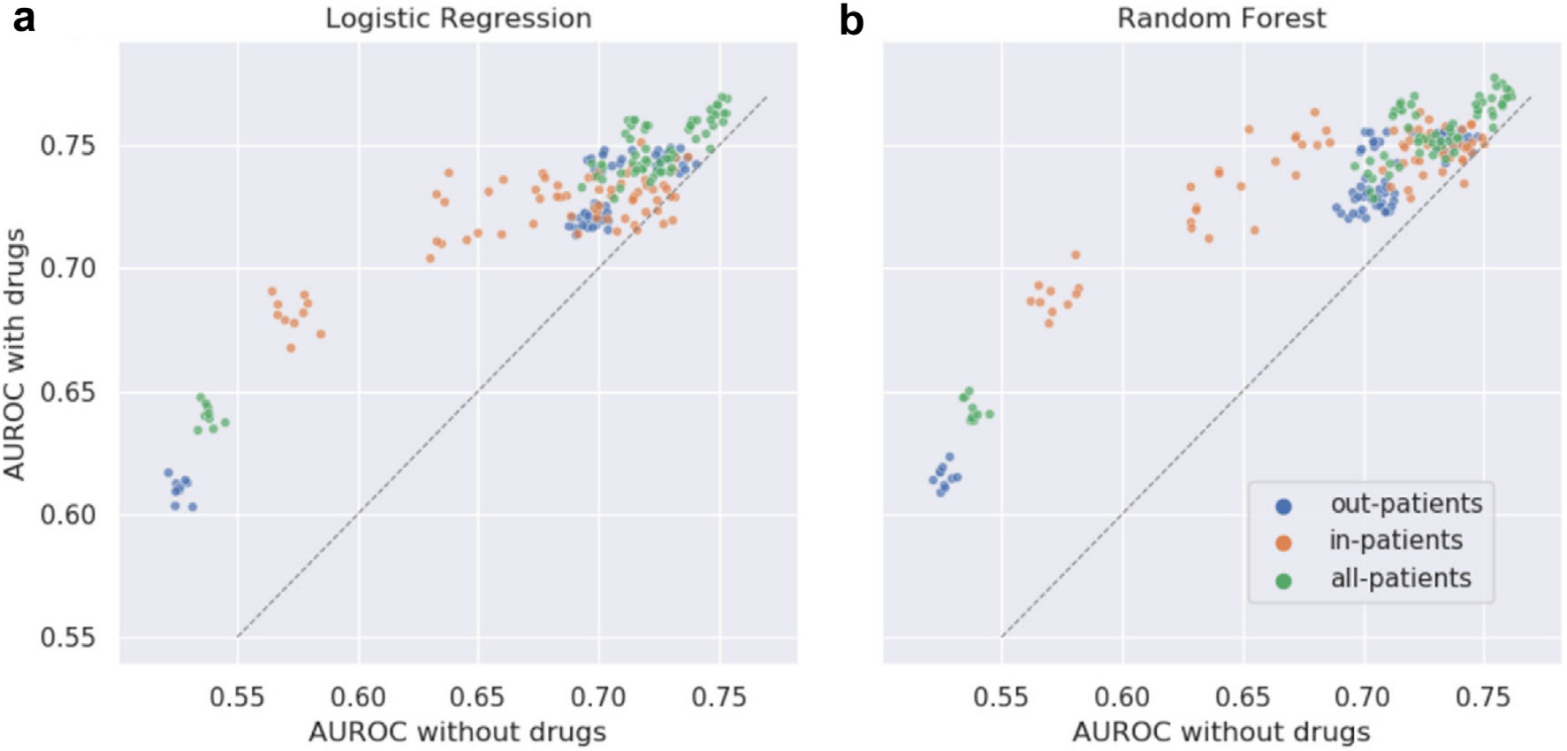
**Importance of drug features in long COVID prediction in all three patient cohorts**. The *x-*coordinate of each point is the AUROC score of a feature category combination and the *y*-coordinate is the score of the same combination but after including drug features. Each cohort is represented by a unique color and has 70 points (seven pairs of feature combinations and ten iterations each). The grey dotted line represents the *x* = *y* line.

For each cohort, we compared performance of the classification models when trained and tested with 15 different combinations of one or more features categories to assess the importance of individual feature categories (Methods: Feature Combinations; Supplementary Figure S2; Supplementary Table S6 (Ablation Study–AUROC, Ablation Study–AUPRC)). The models trained with combinations including drugs achieved higher AUROC (Fig. 4; p-value <0.0001, Wilcoxon signed-rank test).

### Feature importance

**Fig. 5:**
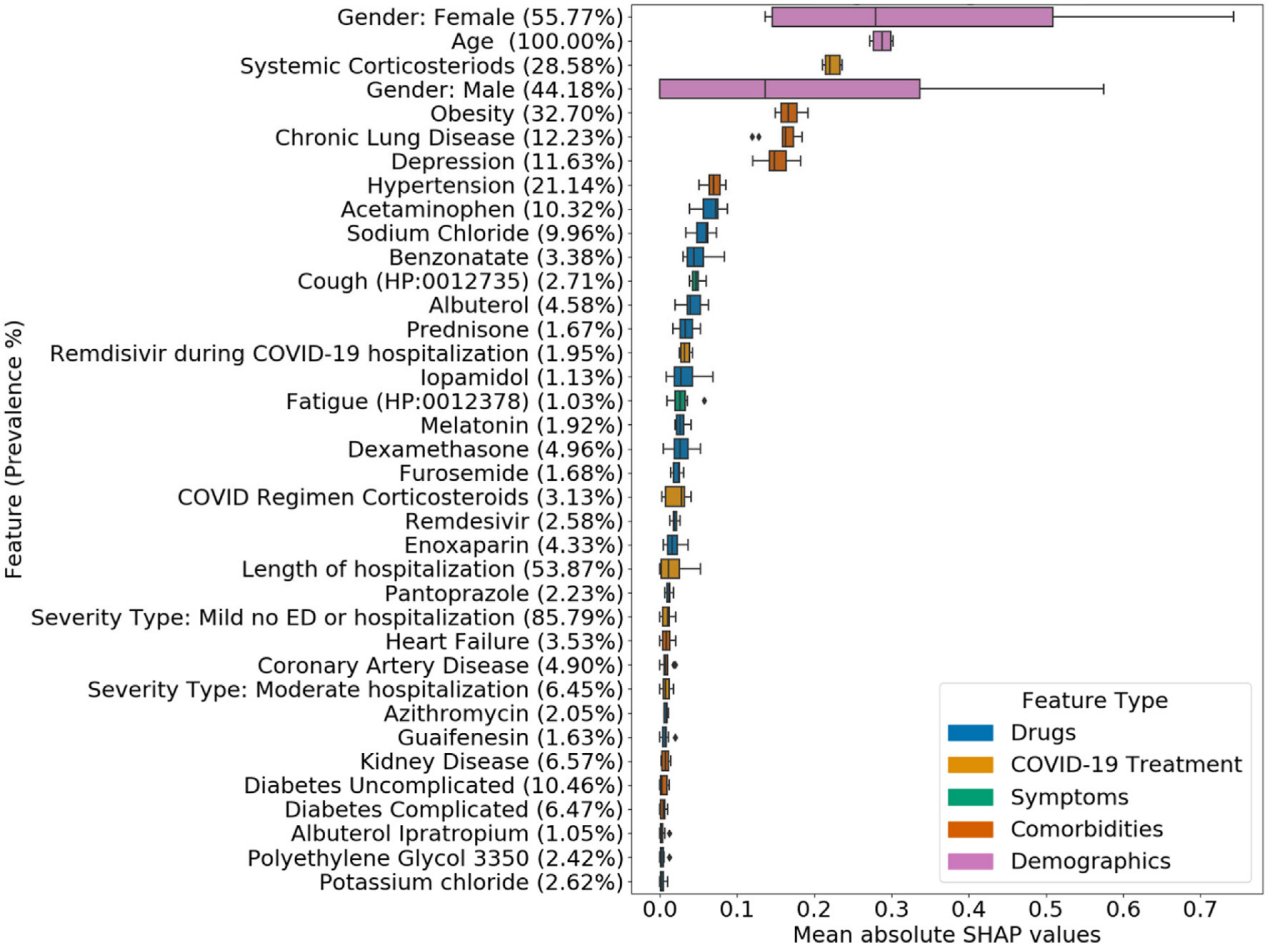
**Importance of features in long COVID prediction models**. Each row (along the *y*-axis) corresponds to a feature. The *x*-axis represents the mean absolute value of SHAP values of the given feature over all test set samples in one iteration. Each boxplot shows the distribution of these mean values for one feature across the iterations (maximum ten) in which it was selected by the Boruta method. The features are sorted in decreasing order of the median of the distribution of their mean absolute SHAP values. In each boxplot, the lower endpoint, the line in the middle, and the higher endpoint denote the first, second, and third quartiles of the distribution. The whiskers span 1.5 times the interquartile range. Diamonds denote values outside this range. The legend displays the mapping between feature category and colour.

We plotted the distribution of the mean absolute SHAP values for every feature selected in at least five of the ten iterations for the LR (Fig. 5) and RF (Supplementary Figure S3) models in the all-patient cohort. Supplementary Table S7 and Supplementary Figure S4 contain the mean SHAP values for each feature, iteration, model, and cohort combination. Across the samples analysed for interpretation of individual predictions, a value of one for the following features influenced the LR model to predict a probability in favour of developing long COVID. These features included age, gender, symptoms such as cough and fatigue during the acute COVID-19 infection, comorbidities such as chronic lung disease, depression, diabetes, kidney disease, and obesity (Supplementary Figure S5).

### Cross-site analysis

The data in N3C is procured from multiple data partner sites across the United States. This data is then harmonised using the OMOP common data model and made available for analysis. Variability in the data obtained from multiple sources has the potential to introduce bias in the results and thus can limit the generalizability of machine learning models trained on multisource data.^39^ We performed a cross-site analysis to gauge the impact of using data from disparate sources on the performance of our models. The patient cohorts in this study contained data from 39 N3C data partners (Fig. 1; Methods: Patient Cohort Definition). For each of these 39 sites, we counted the number of long COVID patients (Supplementary Table S2 (Cross-site Analysis)). The top two contributing data partners (data partners 1 and 2) reported 2,253 and 1,668 long COVID patients, respectively, thereby accounting for 23% of the total number of long COVID samples in our dataset.

We trained models using data from each one of these two data partners (data partners 1 and 2) independently and evaluated the models on data from the remaining sites. Specifically, we built prediction models for each cohort with training data from only data partner 1. We then tested these models with data from the 38 other data partner sites (including data partner 2). We repeated this process for data partner 2.

**Fig. 6:**
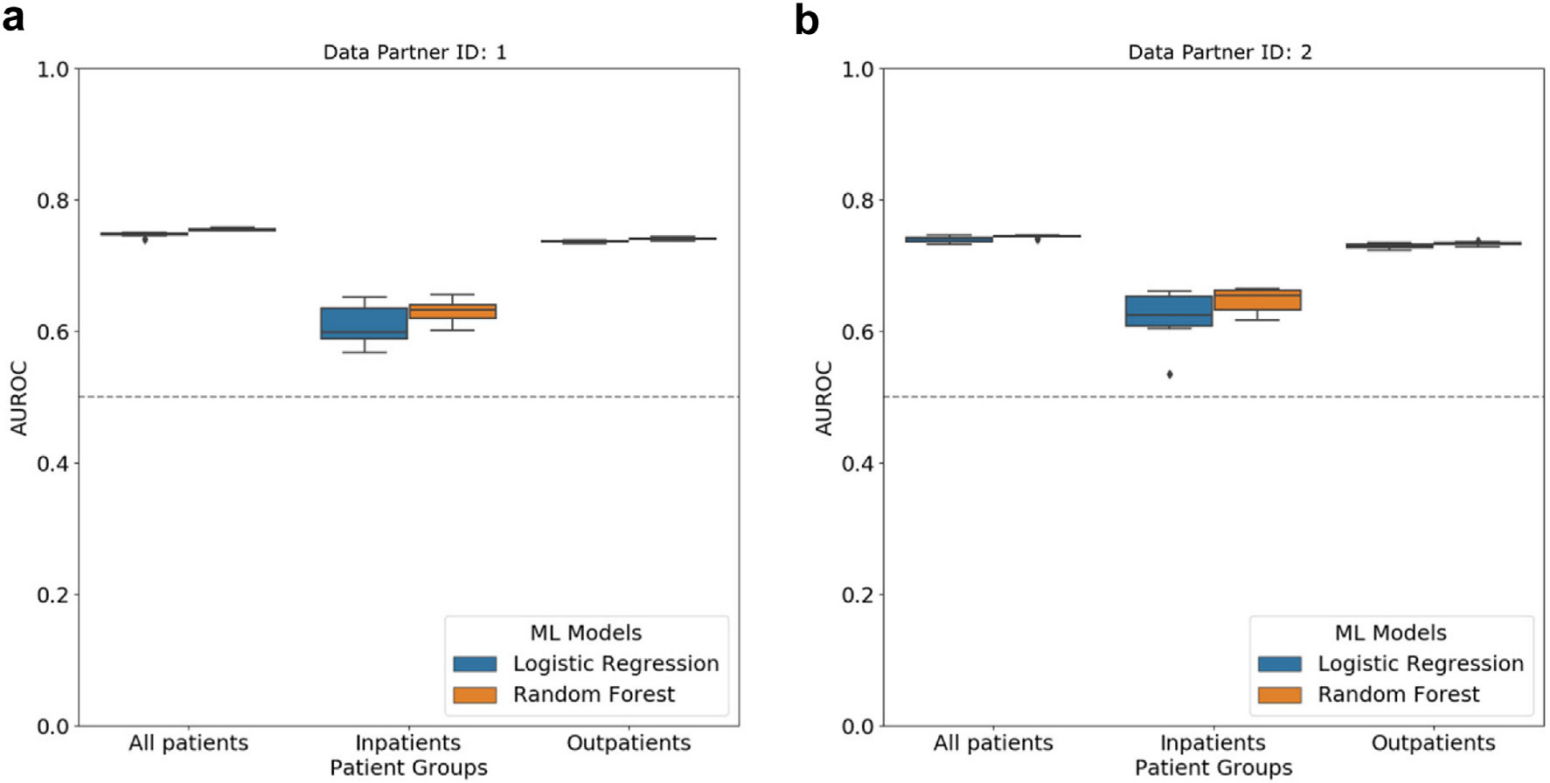
**Performance of long COVID prediction models in cross-site analysis**. Results of cross-site analysis where we train a prediction model on data from only one data partner site and test on data from all other data partners. Distribution of AUROC values from ten iterations of prediction using logistic regression and random forest models when the training dataset comprises data from only **(a)** data partner 1 and **(b)** data partner 2. In each boxplot, the lower endpoint, the line in the middle, and the higher endpoint denote the first, second, and third quartiles of the distribution. The whiskers span 1.5 times the interquartile range. Diamonds denote values outside this range. The grey dotted line represents the expected score of a random predictor in the all-patient cohort.

For both the institutions, LR and RF yielded comparable median AUROC scores between 0.74 (IQR = 0.01) and 0.75 (IQR = 0.00) in the all-patient cohort (Fig. 6; Supplementary Figure S6; Supplementary Table S6 (Cross-site Analysis–AUROC, Cross-site Analysis–AUPRC)).

## Discussion

We predicted long COVID occurrence in COVID-19 patients using EHR data. For this classification task, we used existing comorbidities in patients, symptoms, drugs, and measures of treatment during the acute infection, and patient demographics.

The performance of the RF method was virtually identical to that of the LR (Fig. 3). While AUROC scores were promising in all the experiments, the AUPRC scores were low (Fig. 3), possibly due to the low prevalence of long COVID diagnosis in our cohorts. For example, the RF model had a median AUPRC of 0.04 (IQR = 0.00) in the all-patient cohort (proportion of long COVID patients in cohort = 0.78%). Thus, the model was approximately five times better than a random predictor. Similarly the model outperformed the random predictor by four times in inpatients (2.21%), and was three times better in outpatients (0.67%). Further, we evaluated the same trained models on four additional testing datasets. These datasets contained testing records with varying ratios of positive and negative samples, namely 1:1, 1:2, 1:5, and 1:10. We observed a decline in the AUPRC scores as the proportion of the negative samples in the testing dataset increased (Supplementary Figure S7), while the AUROC scores remained the same (Supplementary Figure S8). The AUPRC metric captures the imbalance in datasets by accounting for both precision and recall. On the other hand, the AUROC metric utilises only the true and false positive rates.

The ablation study helped us compare the value added by each of the feature categories to predicting long COVID. Drugs had the highest predictive information when compared to the other four feature categories (Fig. 4). The exclusion of drugs caused an average decrease of 5.06% in the median AUROC scores of the models in all patients.

Prognosis models for complex conditions such as long COVID are generally built and evaluated using data from the same source.^7^^,^^13^ However, when they are validated externally, though seldom, we find large decrements in performance. Those large decrements often make it unwise to use the models to stratify data from any institutions other than the training data sites. Thus, it is important to assess the performance of models across many data partners, distinct from the training data sites. These observations motivated our novel cross-site analysis (Results: Cross-site Analysis). We trained models on only one data partner (1 or 2) and tested on data from all other partners. We compared the performance of these models with the performance of models trained and tested on data from all partners. With LR trained on EHR from only data partner 1, we observed a decrease in the median AUROC in all patients (decrease = 1.32%, p-value = 0.0002, Wilcoxon Rank Sum test), inpatients (18.92%, 0.0002), and outpatients (0.00%, 0.0002). The differences were similar for the RF model and for both models trained only on data from partner 2. Although the performance decrements were statistically significant (Supplementary Table S8 (Statistical Test Results)), the magnitudes of the decreases were small for all patients and outpatients, and it was noticeable for inpatients. This suggested that data from a single data partner may not sufficiently capture the variation in inpatients’ data across the N3C due to differences in population, care and data quality, and medical practices, thereby impeding the generalizability of the prediction models for inpatients.

We computed SHAP^37^ values of features to analyse their importance in the prediction task (Fig. 6, Supplementary Figures S3–S5). Among the top features, we observed that LR utilised all five types of features whereas RF did not rely on symptoms to make decisions. 21 features appeared in the top 30 features of both the prediction models as well as in the local interpretations of individual test samples. These features included gender, age at COVID index date, severity of the COVID-19 infection, drugs such as acetaminophen (prevalence = 10.32%), albuterol (4.58%), remdesivir during COVID hospitalisation (1.95%), and albuterol ipratropium (1.05%), and comorbidities such as heart failure (3.53%), hypertension (21.14%), obesity (32.70%), kidney disease (6.57%), chronic lung disease (12.23%), diabetes (16.93%), depression (11.63%), and coronary artery disease (4.90%).

There are stark differences in the data sources and types, patient populations, features, and definitions of long COVID considered in prior studies^7^^,^^8^^,^^13^^,^^17^^,^^40^ that characterise long COVID. This diversity limits the extent to which we can directly compare our results to the existing studies. Nevertheless, there is significant support for the features we identified as having high SHAP values. Higher age of patients (feature importance rank for LR = 2, feature importance rank for RF = 14), female sex (LR = 1, RF = 29), and treatment metrics such as severity of COVID-19 infection (LR = 26, RF = 20) and length of hospitalisation (LR = 24, RF = 36) are well-known risk factors of long COVID.^4^^,^^5^^,^^7^^,^^13^^,^^14^ Existing studies validate that chest-pain (LR = 38, RF = 38)^7^ has been found to persist in long COVID patients. Comorbidities such as obesity (LR = 5, RF = 6), anxiety and/or depression (LR = 7, RF = 24), dementia (RF = 8), diabetes (LR = 33, RF = 10), kidney disease (LR = 32, RF = 7), and chronic lung disease (LR = 6, RF = 11) are risk factors for long COVID.^4^^,^^5^^,^^14^^,^^41^ Treatments provided during and after the acute COVID-19 infection such as melatonin (LR = 18)^7^ and polyethylene glycol 3350 (LR = 36)^7^ are also indicative of long COVID.

As in the ablation study, we observed that drugs played a crucial role in classifying patients. Benzonatate and guaifenesin are used for respiratory symptoms. Albuterol is a bronchodilator used for asthma, wheezing, and respiratory symptoms caused by viral infections. These drugs have been used on a large scale for symptomatic relief in COVID-19 infections.^42^ Dexamethasone and prednisone are corticosteroid medications used to decrease the immune response, which might have detrimental effects, in COVID-19.^43^^,^^44^ While the role of corticosteroids in long COVID treatment is under investigation, these agents may reduce the symptoms and some of the immunological alterations present in long COVID.^45^ Patients hospitalised with moderate to severe COVID-19 infection have been treated with corticosteroids and remdesivir.^45^, ^46^, ^47^, ^48^, ^49^, ^50^ While melatonin is a mild sleep aid, a study found decreased mortality in patients treated with it.^51^ Melatonin has also been proposed as an adjuvant treatment in COVID-19^52^^,^^53^ and for treating long COVID.^54^ Enoxaparin is an anticoagulant medication used to prevent and treat deep vein thrombosis. In COVID-19, enoxaparin used prophylactically has been associated with a significant reduction of mortality.^55^ Antihistamines and azithromycin have been proposed for the early treatment of COVID-19 for severity reduction and long COVID. While the exact mechanism of antihistamines causing the antiviral effect remains unclear, studies hypothesise that antihistamines inhibit the proinflammatory cytokine storm and virus binding in COVID-19.^56^, ^57^, ^58^, ^59^ The use of these drugs suggests that the corresponding comorbidities and symptoms might increase the risk of long COVID.

We conclude this section with the limitations of our study. First, as reported by Pfaff et al.,^7^ the laboratory data are sparsely represented in the N3C Enclave. The proportion of missing values in the harmonised measurements ranged from 68.25% to 99.99%. Therefore, we did not include laboratory test values measured during the acute COVID-19 infection as features. Furthermore, three years after the emergence of COVID-19, information about the different variants of SARS-CoV-2 and COVID-19 vaccinations administered to patients may play an important role in the development of long COVID in COVID-19 patients.^4^ However, N3C Enclave lacked this data at the time of performing this study.

Second, EHRs are biased towards patients seeking health care or having health insurance at institutions that have partnered with the N3C. Moreover, the lack of data about a patient in N3C cannot be definitively associated with the absence of the disease condition. The demographic information of the patient cohorts in Supplementary Table S1 shows that a higher number of people in the age group 51–80 years were hospitalised. The cohorts also had a significantly higher representation of patients whose race is ‘white’ while males were slightly over represented than females. Thus, the cohorts do not guarantee a holistic representation of all COVID-19 patients.

Finally, literature estimates of the proportion of COVID-19 patients who have long COVID range from 10 to 70%^6^^,^^14^ and are much larger than the approximate 0.3% of long COVID patients in our cohorts. The American version of the 2022 ICD-10-CM diagnosis code U09.9, which we used to label long COVID patients, was released on October 1, 2021. Prior to this date, patients were diagnosed using the more general ICD-10-CM code B94.8,^60^ which includes all forms of sequelae of unspecified infectious and parasitic diseases not already encapsulated by the ICD-10-CM codes in the range B90–B94.^60^ Since our study considered longitudinal data starting from January 1, 2018, it is likely that some of the early long COVID patients may have been diagnosed with the ICD-10-CM code B94.8. Besides, the adoption of the U09.9 code for long COVID diagnosis by the health institutions has been slow.^12^ Hence, we analysed the EHRs of patients labelled incorrectly as long COVID patients by our prediction models, i.e., false positives (Supplementary Method: B94.8 analysis). 8.78% of them were diagnosed with the ICD-10-CM code B94.8. There is no data available in the N3C on the nuances of the usage of U09.9 code such as the number of long COVID patients seeking clinical referral being accounted for in this code. McGrath et al. studied the adoption and use of this code in the US using commercial insurance claims data and found that 50.9% of the long COVID data came from outpatient settings whereas 6.8% was from inpatients. However, 37.2% of the U09.9 diagnosis could not be traced back to categorizable sources.^21^

### Conclusion

We predicted the occurrence of long COVID in COVID-19 patients using their EHRs from the N3C Enclave. We leveraged the symptoms experienced by COVID-19 patients, the drugs ordered for them, and treatment details (if hospitalised) during their SARS-CoV-2 infection, their demographic information, and comorbidities to implement long COVID predictors based on two classical ML models — logistic regression and random forest. The models performed on par with each other across different patient cohorts and feature combinations. We validated the generalizability of the predictors through cross-site analysis. We computed feature importance values to explain the predictions of the classifiers. Given the lack of well-defined symptoms and attributes to diagnose long COVID, healthcare institutions and clinicians could leverage the proposed computational methods to identify COVID-19 patients who may be at risk of developing long COVID. These patients could then be advised on the need for follow-up or preventive measures to alleviate or prevent the possible manifestation of long COVID.^4^

Training models using deep learning is a key direction for future research. Including vaccination status and socio-economic factors can enrich our datasets. We may also impose a lower bound constraint on the gap between the end of the acute infection phase and the long COVID diagnosis. These considerations may lead to more powerful long COVID predictors in the future.

## Contributors

Conceptualization: BA, PNR, JTR, TMM.

Methodology: BA, HB, EC, JTR, JJL, TJC, BJL, KJW, CCA, GV, AEW, TMM.

Clinical expertise: CCA, AEW, PNR.

Funding acquisition: TMM.

Supervision: PNR, JTR, TMM.

Writing—original draft: BA, TMM.

Writing—review & editing: HB, EC, JJL, TJC, BJL, KJW, CCA, GV, AEW, PNR, JTR, TMM.

All authors read and approved the final version of the manuscript.

BA, JTR, and TMM verified the underlying data.

## Data sharing statement

All experiments and analyses described in this study were conducted using de-identified (level two) EHRs data in the NCATS N3C Data Enclave (https://ncats.nih.gov/n3c/) which is restricted to registered N3C users. Researchers can apply for access to the N3C Enclave as described in https://ncats.nih.gov/n3c/about/applying-for-access. All curated patient cohorts, results, analyses outputs, visualisations, and source code are available in the project with Data User Request RP-6DC499 “Prediction of Symptoms Associated with Long COVID”.

## Declaration of interests

J Loomba received consulting fees from Axle Informatics as a subject matter expert for RadxUp Long COVID computational challenge (L3C). The other authors declare no competing interests.

## Appendix A Supplementary data

Supplementary Table S8

Consortial authors
