# Supplementary material for: Predictive models of long COVID
Source: eBioMedicine. 2023 Sep 4;96:104777. doi: 10.1016/j.ebiom.2023.104777 (PMC10494314; doi:10.1016/j.ebiom.2023.104777)
Supplement: Supplement 1 [file mmc1.docx]

Predictive Models of Long COVID

SUPPLEMENTARY MATERIAL

**TABLE OF CONTENTS**

[Supplementary Method: Ablation study dataset construction](#_sqer9kdi6sjo) **1**

[Supplementary Method: SHAP interpretation analysis](#_g6fh8l7tz0df) **1**

[Supplementary Method: B94.8 analysis](#_psl18u4vqrbx) **1**

[Supplementary Method: Implementation](#_thjnd7y0omxx) **1**

[Supplementary Figure S1: ROC curves by model, by patient cohort](#_42zw5hvj8vfg) **3**

[Supplementary Figure S2: Median AUROC and AUPRC scores of ablation study](#_52ho03w7q0ci) **4**

[Supplementary Figure S3: Importance of features in the prediction of long COVID using random forest.](#_5n5adzjuqgbj) **5**

[Supplementary Figure S4: Feature importance by model, by patient cohort](#_g9dt4wpydxwe) **6**

[Supplementary Figure S5: Local explanation of long COVID prediction using Logistic Regression.](#_xl0s6dkexs0s) **7**

[Supplementary Figure S6: AUPRC scores of long COVID prediction models in cross-site analysis.](#_r11sawxp5a42) **8**

[Supplementary Figure S7: AUPRC scores of long COVID models with varying ratios of positive and negative samples.](#_txu8qb1u1tyg) **9**

[Supplementary Figure S8: AUROC scores of long COVID models with varying ratios of positive and negative samples.](#_15cc60ctq5c) **10**

SUPPLEMENTARY METHODS

#

# Supplementary Method: Ablation study dataset construction

In ablation study, each of the 15 combinations of feature categories had three separate datasets — one for each cohort. To construct these 45 datasets, we first computed the independent datasets for each of the five feature categories. We then created an overall dataset comprising all the features by including only those patients that were present in at least one of the five feature category-specific datasets. This dataset contained data from all the 39 data partners that used the ICD-10-CM code U09*.*9. We performed this computation separately for all patients, inpatients, and outpatients. Finally, to generate the 42 remaining datasets, we selected the corresponding subsets of features.

# Supplementary Method: SHAP interpretation analysis

In one (out of ten) randomly selected iteration of the logistic regression (LR) classification on all patients, the dataset contained 438,741 (3,401 positive and 435,340 negative) samples. We traversed the precision-recall curve and selected a prediction score of 0.91 as the threshold, since the number of test samples whose scores were at least as large as this cutoff (the positive predictions) was almost equal to the number of positive examples in the test dataset. Using this threshold to make predictions, we analysed the SHAP values of random test samples from true positives, false positives, true negatives, and false negatives sets.

# Supplementary Method: B94.8 analysis

We randomly selected one of the ten iterations of classification by the random forest (RF) model on the all-patient cohort. This dataset consisted of 438,398 samples with 3,350 positive and 435,048 negative samples. To label the probabilities computed by the RF model, we traversed the precision-recall curve and selected the threshold that yielded an almost equal number of positive and negative predictions as in the original test dataset. In this way, using the threshold of 0.83 we labelled the outputs of the classification model to get 3,353 positive and 435,045 negative predictions. The 3,353 positive predictions contained 3,088 false positives. We examined all the disease condition records after the COVID-19 index date of these 3,088 patients and looked for patients diagnosed with the ICD-10-CM code B94.8.

# Supplementary Method: Implementation

All methods and experiments presented in this manuscript were implemented using Python v3.6.10. We used boruta_py v0.3 for feature selection, pyspark v3.2.1-palantir.5.1 for dataset curation and preprocessing, and scikit-learn v0.24.2 for dataset splits, LR, RF, cross validation, hyperparameter search, and evaluation. Further, we used shap v0.39.0 to compute feature importance. Finally, we used matplotlib v2.2.4 and seaborn v0.11.2 for all visualisations. The data and code used in this study is available under the Data Use Request RP-6DC499 - “Prediction of Symptoms Associated with Long COVID” in the N3C Enclave.

SUPPLEMENTARY FIGURES


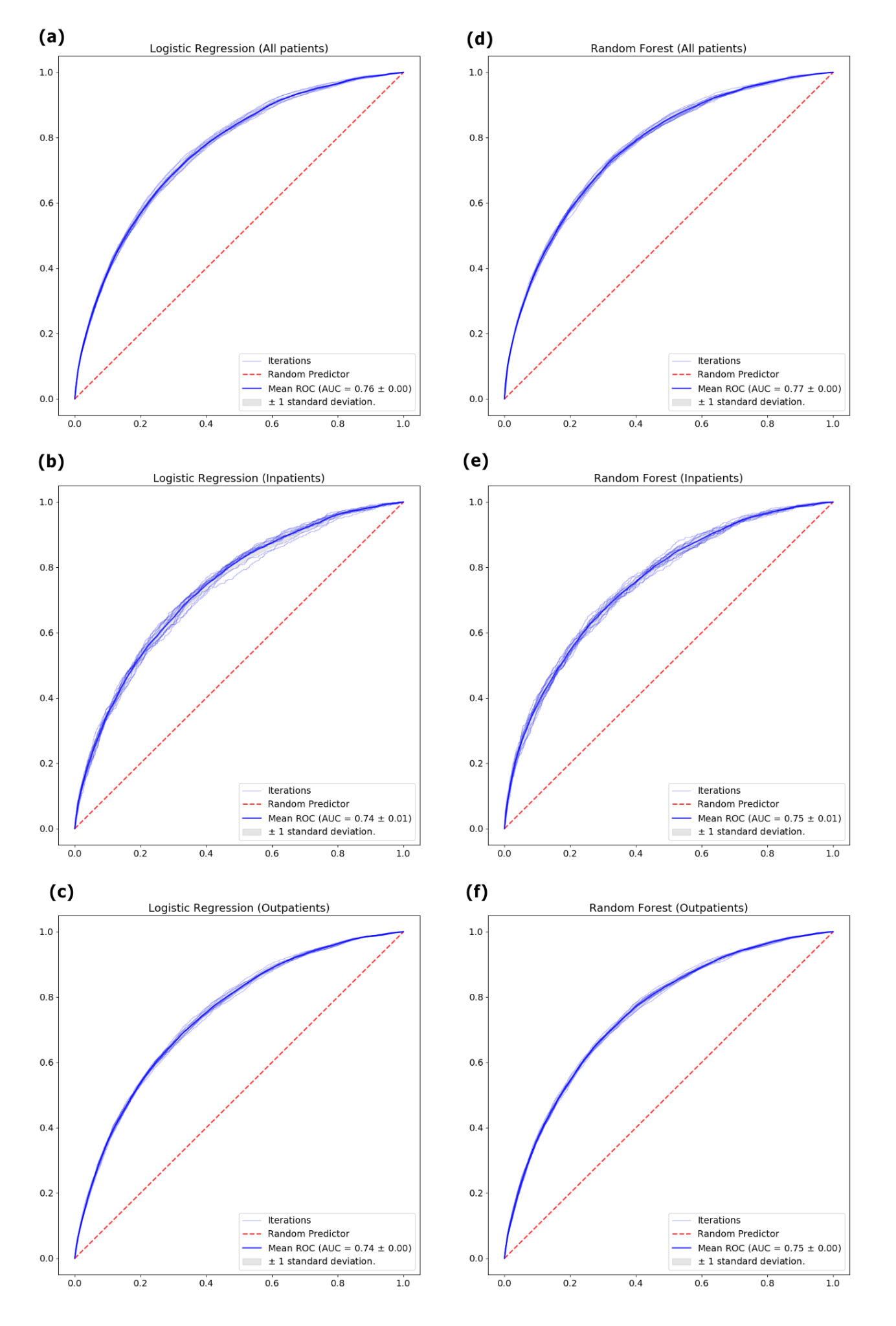


# Supplementary Figure S1: ROC curves by model, by patient cohort

Receiver operating characteristic (ROC) curves for logistic regression and random forest models for all ten iterations, the average area under receiver operating characteristic (AUROC), and the standard deviation for **(a)** all patients, **(b)** inpatients, and **(c)** outpatients. ROC curves for random forest model for all ten iterations, the average AUROC, and the standard deviation for **(d)** all patients, **(e)** inpatients, and **(f)** outpatients.

#
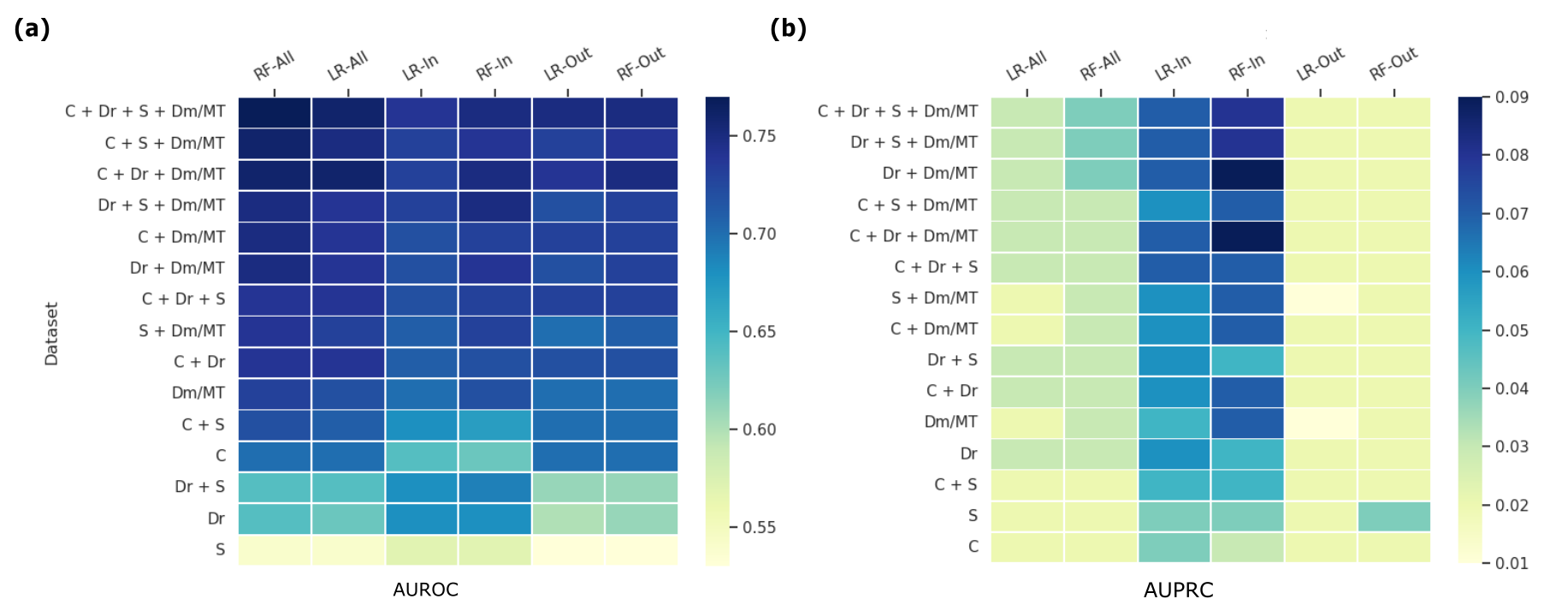
Supplementary Figure S2: Median AUROC and AUPRC scores of ablation study

Median **(a)** AUROC and **(b)** AUPRC scores over ten iterations of ablation study using 15 different combinations of one or more feature categories: comorbidities (C), drugs (Dr), symptoms (S), demographics (Dm), and measures of COVID-19 treatment (MT). Each row represents a feature combination. Each column corresponds to one model-cohort combination. The combinations are sorted in decreasing order of the corresponding random forest median AUROC scores for the all-patient cohort. Abbreviations: LR, logistic regression; RF, random forest; All, all patients; In, inpatients; and Out, outpatients.


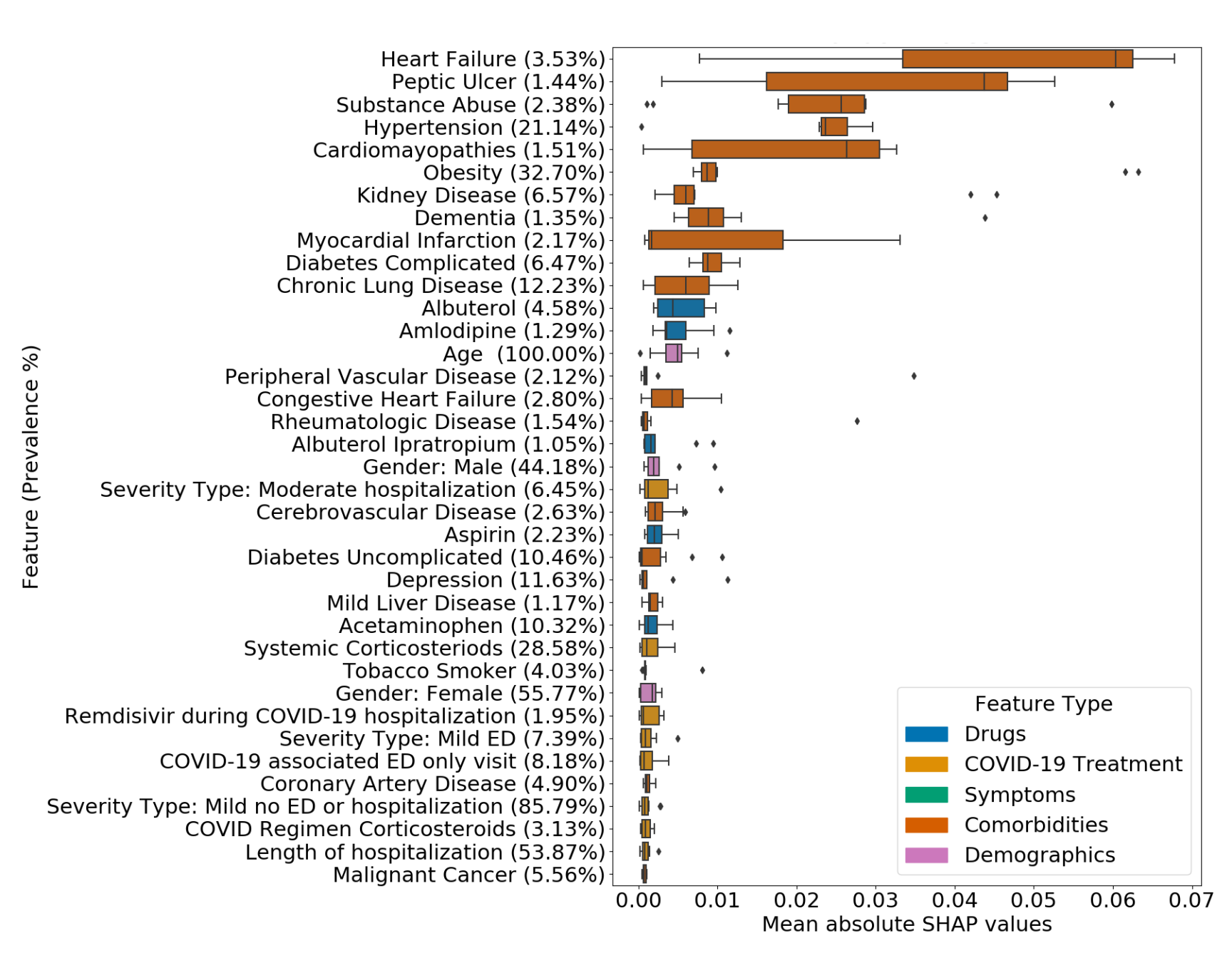


# Supplementary Figure S3: Importance of features in the prediction of long COVID using random forest.

Each row (along the *y*-axis) corresponds to a feature. The *x*-axis represents the mean absolute value of SHAP values of the given feature over all test set samples in one iteration. Each boxplot shows the distribution of these mean values for one feature across the iterations (maximum ten) in which it was selected by the Boruta method. The features are sorted in decreasing order of the median of the distribution of their mean absolute SHAP values. In each boxplot, the lower endpoint, the line in the middle, and the higher endpoint denote the first, second, and third quartiles of the distribution. The whiskers span 1.5 times the interquartile range. Diamonds denote values outside this range. The legend displays the mapping between feature category and colour.


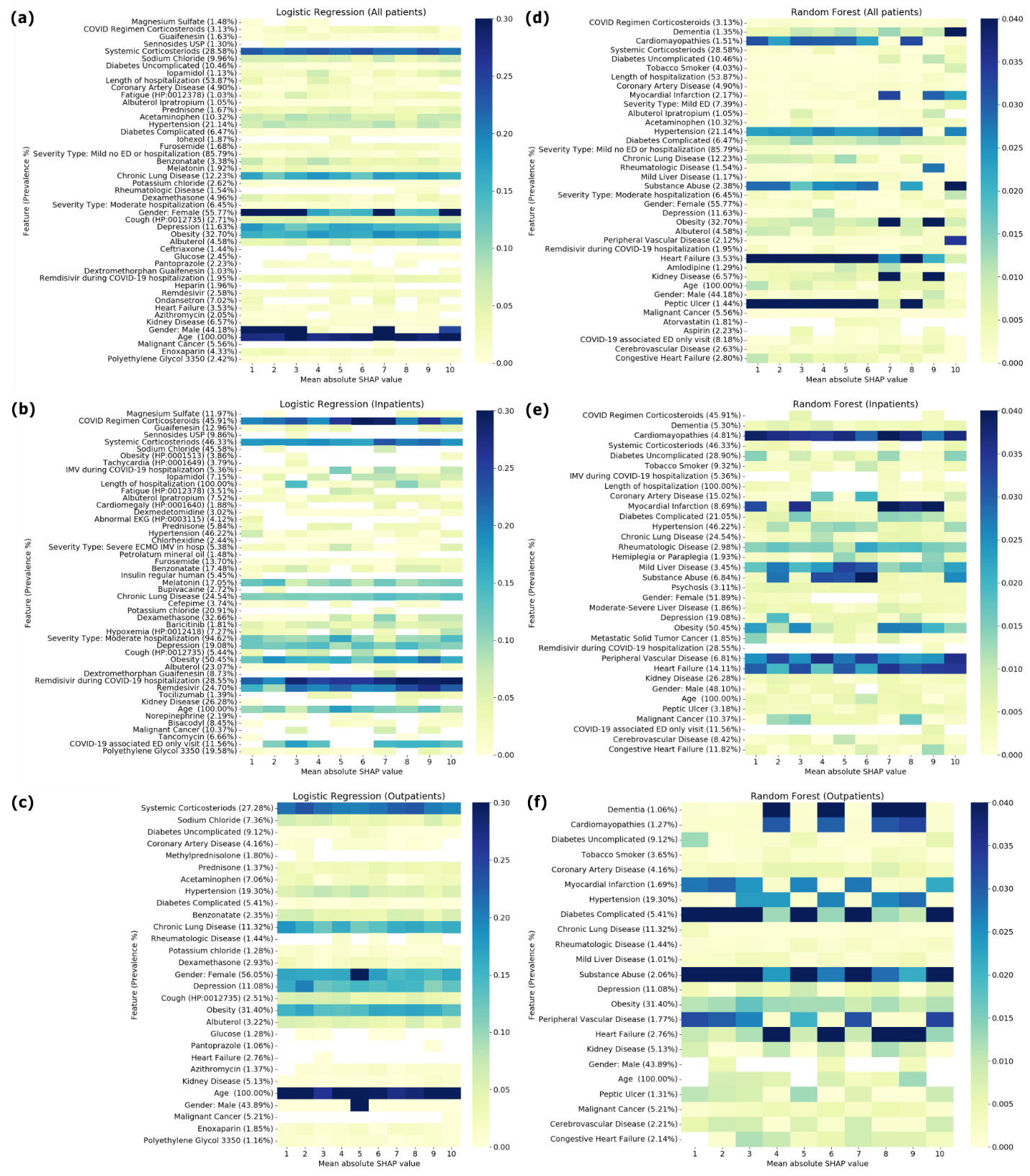


# Supplementary Figure S4: Feature importance by model, by patient cohort

An overview of the features selected (*y*-axis) in every iteration (*x*-axis) of long COVID prediction along with the importance of the selected features in the form of a heat map. Each cell represents the mean absolute value of SHAP values of the given feature over all test set samples in the given iteration. A white cell in these plots denotes that the feature on the y-axis was not selected by the Boruta method for the corresponding iteration on the *x*-axis. **(a)** All patients - Logistic Regression **(b)** Inpatients - Logistic Regression **(c)** Outpatients - Logistic Regression **(d)** All patients - Random Forest **(e)** Inpatients - Random Forest **(f)** Outpatients - Random Forest.


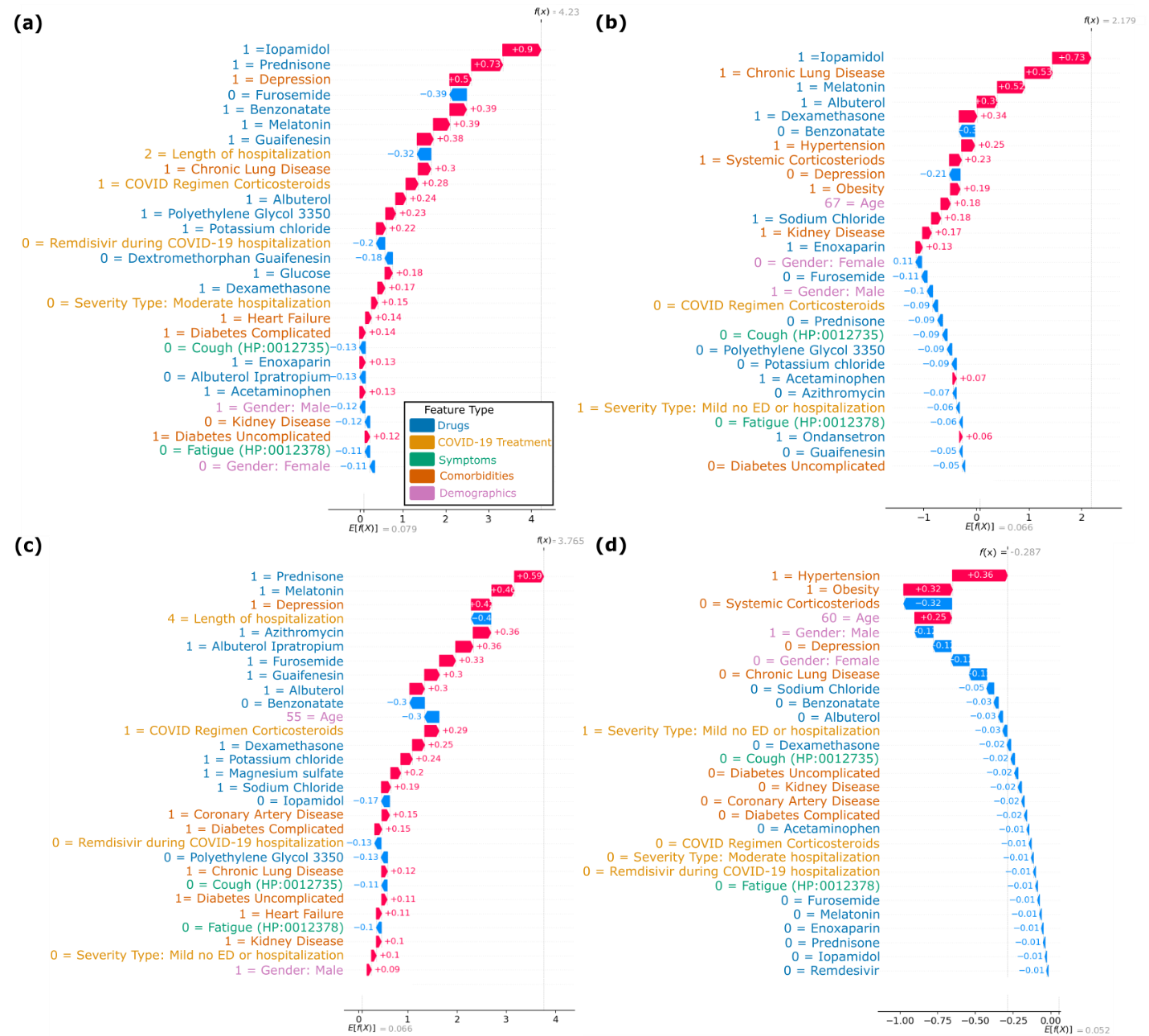


# Supplementary Figure S5: Local explanation of long COVID prediction using Logistic Regression.

Waterfall plot showing the SHAP interpretations of the logistic regression predictions for four randomly selected samples in the **(a)** True Positives **(b)** False Negatives **(c)** False Positives and **(d)** True Negatives sets respectively. The feature values are binary for all features, except age (number of years) and length of stay in hospital (number of days). The legend in **(a)** denotes the categories of the features for all four subfigures. For each of the samples, the figure denotes the features along with their raw values in the *y-*axis, and the contribution of the feature in making the prediction (length of the red/blue bar) in the *x-*axis. The grey vertical line labelled as ‘$f(x)$’ reports the predicted value of the model for the given input sample. ‘$E[f(x)]$’ on the *x*-axis is the expected value of the target variable, i.e., the mean of all predictions in the test dataset. The sum of all SHAP values of individual features is equal to the difference between $E[f(x)]$ and $f(x)$. In the four plots, $E[f(x)]$ is lower than 0.5 (neutral prediction). Positive (negative) SHAP values indicate the influence of the feature value in increasing (decreasing) the predicted probability of long COVID. Features represented with red SHAP values push the model toward a positive prediction for long COVID, whereas ones with blue values favour negative label prediction.

**
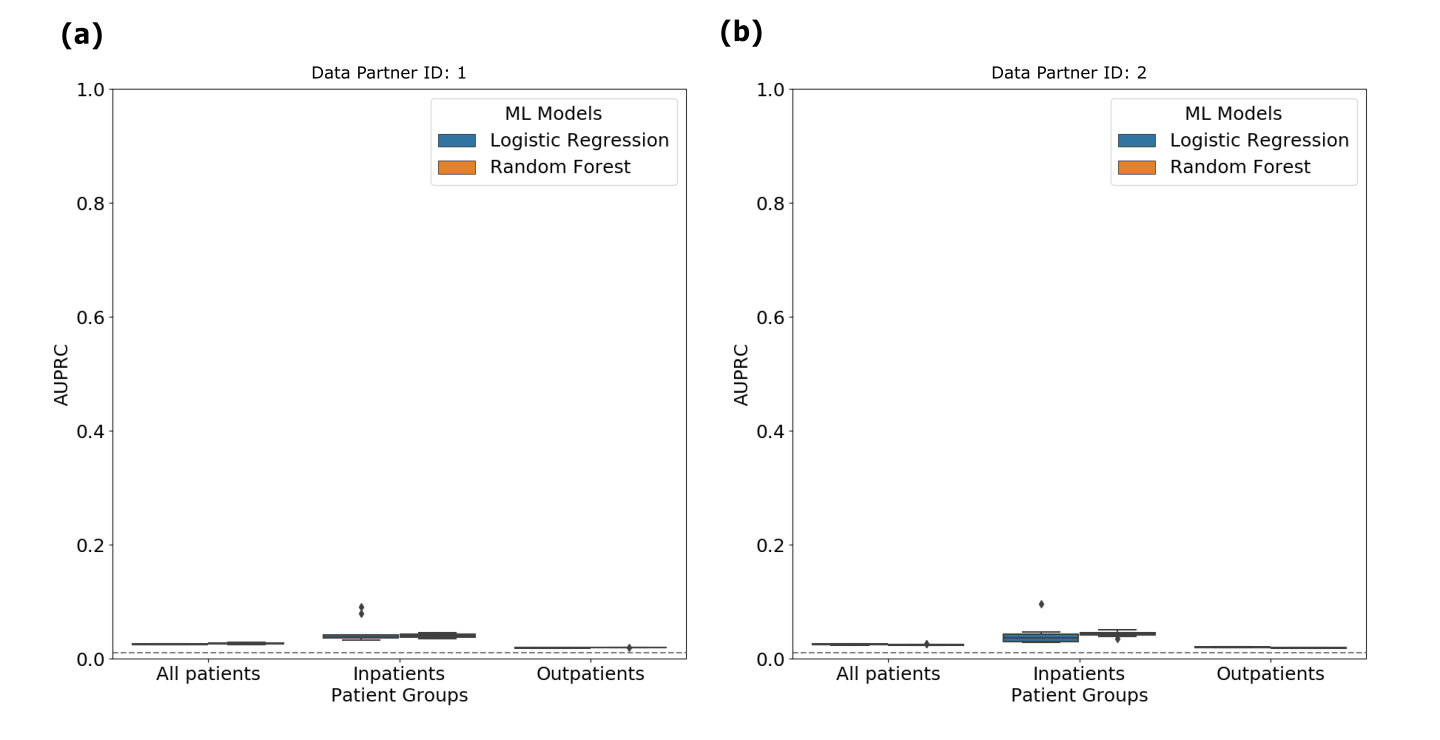
**

# Supplementary Figure S6: AUPRC scores of long COVID prediction models in cross-site analysis.

AUPRC scores of prediction models where we train a prediction model on data from only one data partner site, then test on data from all other data partners. Distribution of AUPRC values from ten iterations of prediction using logistic regression and random forest models when the training dataset comprised data from only **(a)** data partner 1 and **(b)** data partner 2. In each boxplot, the lower endpoint, the line in the middle, and the higher endpoint denote the first, second, and third quartiles of the distribution. The whiskers span 1.5 times the interquartile range. Diamonds denote values outside this range. The grey dotted line represents the expected score of a random predictor in the all-patient cohort.


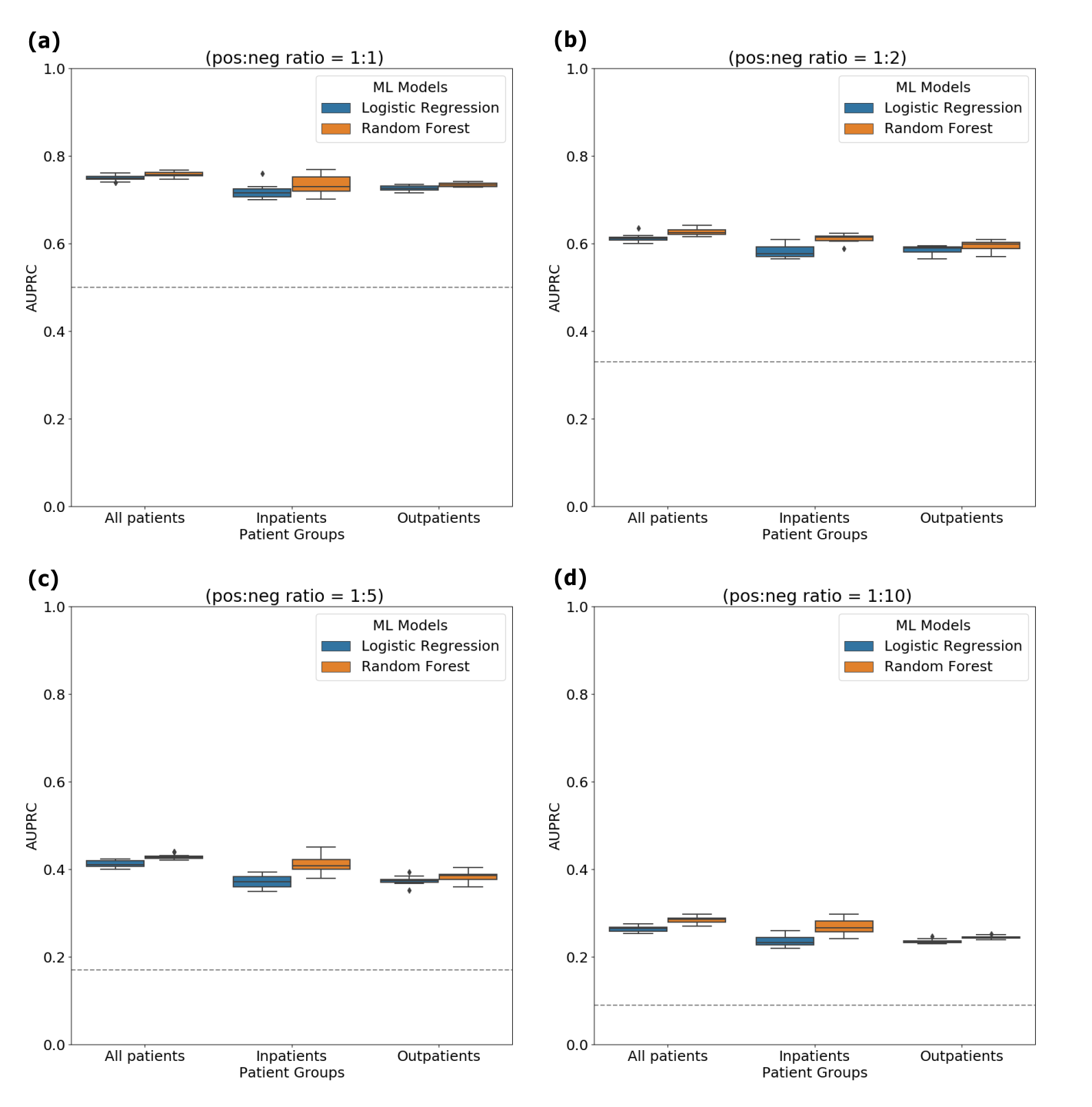


# Supplementary Figure S7: AUPRC scores of long COVID models with varying ratios of positive and negative samples.

Distribution of AUPRC scores for long COVID classification using logistic regression and random forest models for all patients, inpatients, and outpatients when the test set is sampled to contain positive and negative samples in the ratio of **(a)** 1:1 **(b)** 1:2 **(c)** 1:5 and **(d)** 1:10. The AUPRC scores decrease as the number of negative samples increases in the testing dataset. The models were trained using an equal number of positive and negative samples in all four cases. In each boxplot, the lower endpoint, the line in the middle, and the higher endpoint denote the first, second, and third quartiles of the distribution. The whiskers span 1.5 times the interquartile range. Diamonds denote values outside this range. The grey dotted line represents the expected score of a random predictor in the all-patient cohort.


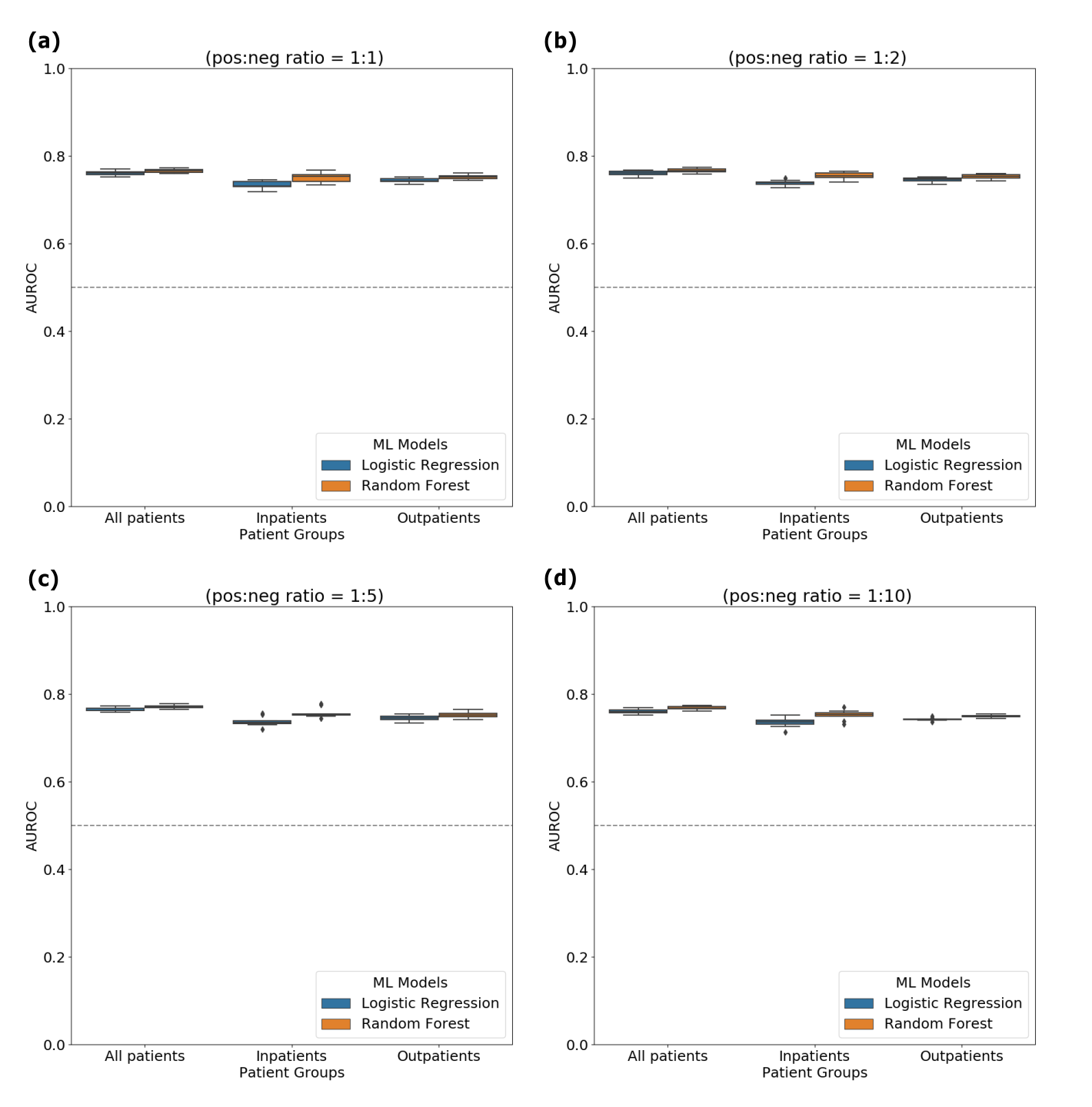


# Supplementary Figure S8: AUROC scores of long COVID models with varying ratios of positive and negative samples.

Distribution of AUROC scores for long COVID classification using logistic regression and random forest models for all patients, inpatients, and outpatients when the test set is sampled to contain positive and negative samples in the ratio of **(a)** 1:1 **(b)** 1:2 **(c)** 1:5 and **(d)** 1:10. The AUROC scores do not vary with increase in negative samples in the testing dataset. The models were trained using an equal number of positive and negative samples in all four cases. In each boxplot, the lower endpoint, the line in the middle, and the higher endpoint denote the first, second, and third quartiles of the distribution. The whiskers span 1.5 times the interquartile range. Diamonds denote values outside this range. The grey dotted line represents the expected score of a random predictor in the all-patient cohort.

SUPPLEMENTARY TEXT

# Abbreviations

All the abbreviations used in this study are listed below

1. COVID-19: Coronavirus Disease 2019
2. COVID: Coronavirus Disease
3. PASC: Post-acute sequelae of Severe acute respiratory syndrome coronavirus 2 infection
4. SARS-CoV-2: Severe acute respiratory syndrome coronavirus 2
5. WHO: World Health Organization
6. ICD10-CM: International Classification of Diseases, Tenth Revision, Clinical Modification
7. EHR: Electronic Health Record
8. N3C: National COVID Cohort Collaborative
9. LR: Logistic Regression
10. RF: Random Forest
11. ML: Machine Learning
12. ZIP: Zone Improvement Plan
13. RT-PCR: Reverse transcription-polymerase chain reaction
14. CDC: Centers for Disease Control and Prevention
15. OMOP: Observational Medical Outcomes Partnership
16. SNOMED: Systemized Nomenclature of Medicine
17. OMOP2OBO: Mapping between OMOP and Open Biomedical Ontologies
18. HPO: Human Phenotype Ontology
19. IMV: Intermittent Mandatory Ventilation
20. ECMO: Extracorporeal membrane oxygenation
21. AUROC: Area under receiver operating characteristic curve
22. AUPRC: Area under precision-recall curve
23. IQR: inter-quartile range
24. SHAP: SHapely Additive exPlanations
25. NCATS: National Center for Advancing Translational Sciences
26. HIPAA: Health Insurance Portability and Accountability Act
27. NIH: National Institutes of Health
