# Supplementary material for: Predictive models of long COVID
Source: eBioMedicine. 2023 Sep 4;96:104777. doi: 10.1016/j.ebiom.2023.104777 (PMC10494314; doi:10.1016/j.ebiom.2023.104777)
Supplement: Consortial authors [file mmc10.docx]

National COVID Cohort Collaborative (N3C) consortium

| First Names | Surname | ORCID numeric |
| --- | --- | --- |
| Christopher | Chute | 0000-0001-5437-2545 |
